# Supplementary material for: Intra-subject sample size effects in plantar pressure analyses
Source: PeerJ. 2021 Jun 24;9:e11660. doi: 10.7717/peerj.11660 (PMC8236230; doi:10.7717/peerj.11660)
Supplement: Supplemental Information 1 [file peerj-09-11660-s001.docx]

Electronic Supplementary Material for

**Sample size effects plantar pressure analyses**

J. McClymont, R. Savage, T. C. Pataky, R. H. Crompton, J. Charles and K. T. Bates

This document contains:

Supplementary Figures 1-8 (Figures S1-8).

Supplementary Tables (Tables S1-2)


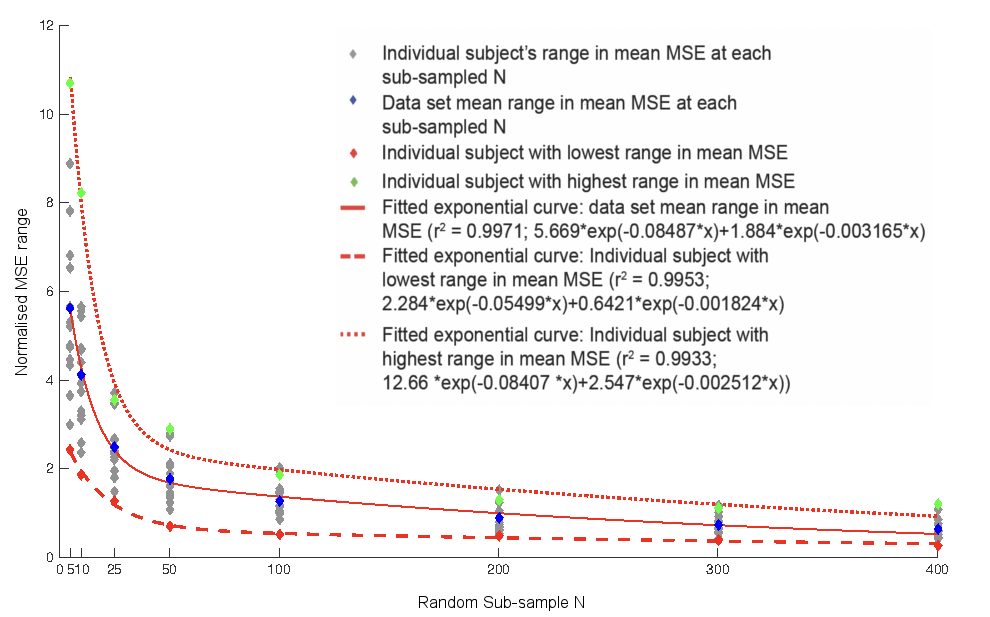


**Figure S1.** The relationship between subsample *N* and the range in mean MSE at each subsample given by 1000 randomly generated subsamples at a walking speed of 1.1ms-1. This relationship is well described by exponential curves in all subjects, which are plotted here for the overall mean of all subjects and the subjects with the highest (7) and lowest (13) overall MSE.


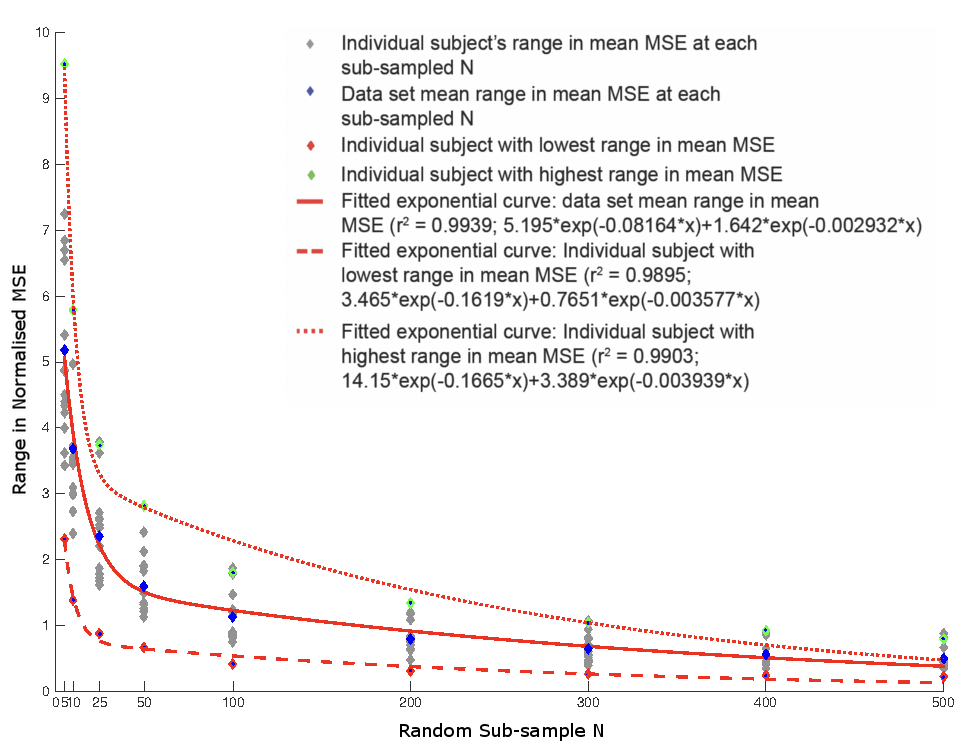


**Figure S2.** The relationship between subsample *N* and the range in mean MSE at each subsample given by 1000 randomly generated subsamples at a walking speed of 1.5ms-1. This relationship is well described by exponential curves in all subjects, which are plotted here for the overall mean of all subjects and the subjects with the highest (7) and lowest (13) overall MSE.


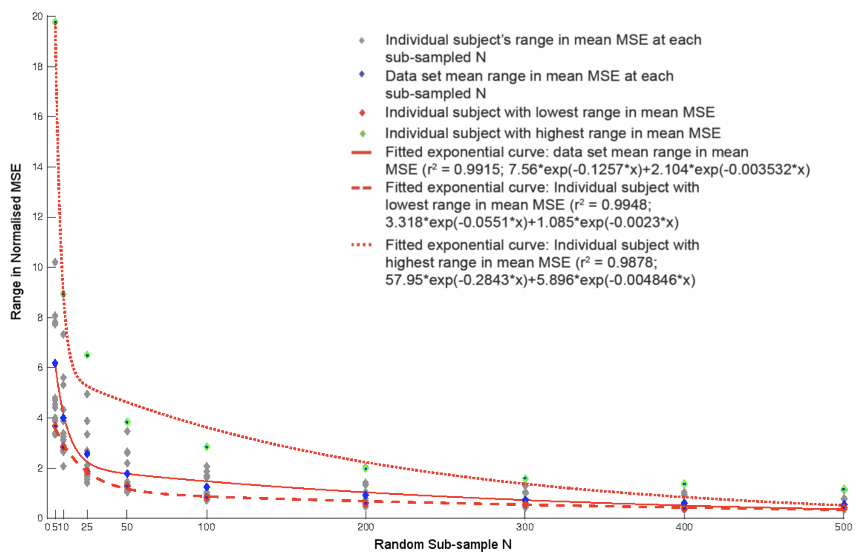


**Figure S3.** The relationship between subsample *N* and the range in mean MSE at each subsample given by 1000 randomly generated subsamples at a walking speed of 1.7ms-1. This relationship is well described by exponential curves in all subjects, which are plotted here for the overall mean of all subjects and the subjects with the highest (7) and lowest (13) overall MSE.


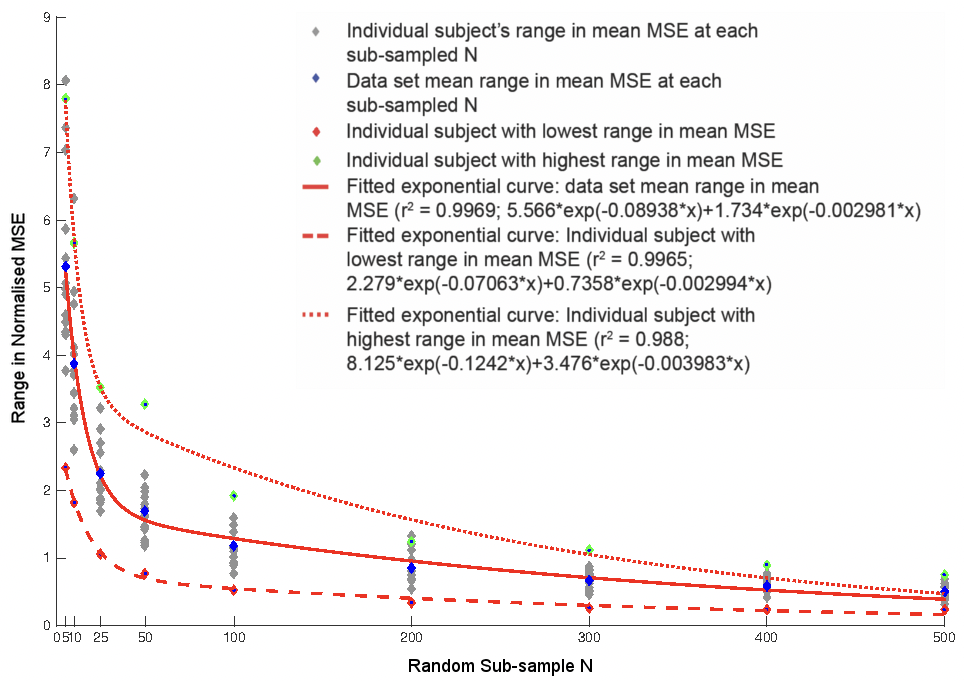


**Figure S4.** The relationship between subsample *N* and the range in mean MSE at each subsample given by 1000 randomly generated subsamples at a walking speed of 1.9ms-1. This relationship is well described by exponential curves in all subjects, which are plotted here for the overall mean of all subjects and the subjects with the highest (7) and lowest (13) overall MSE.


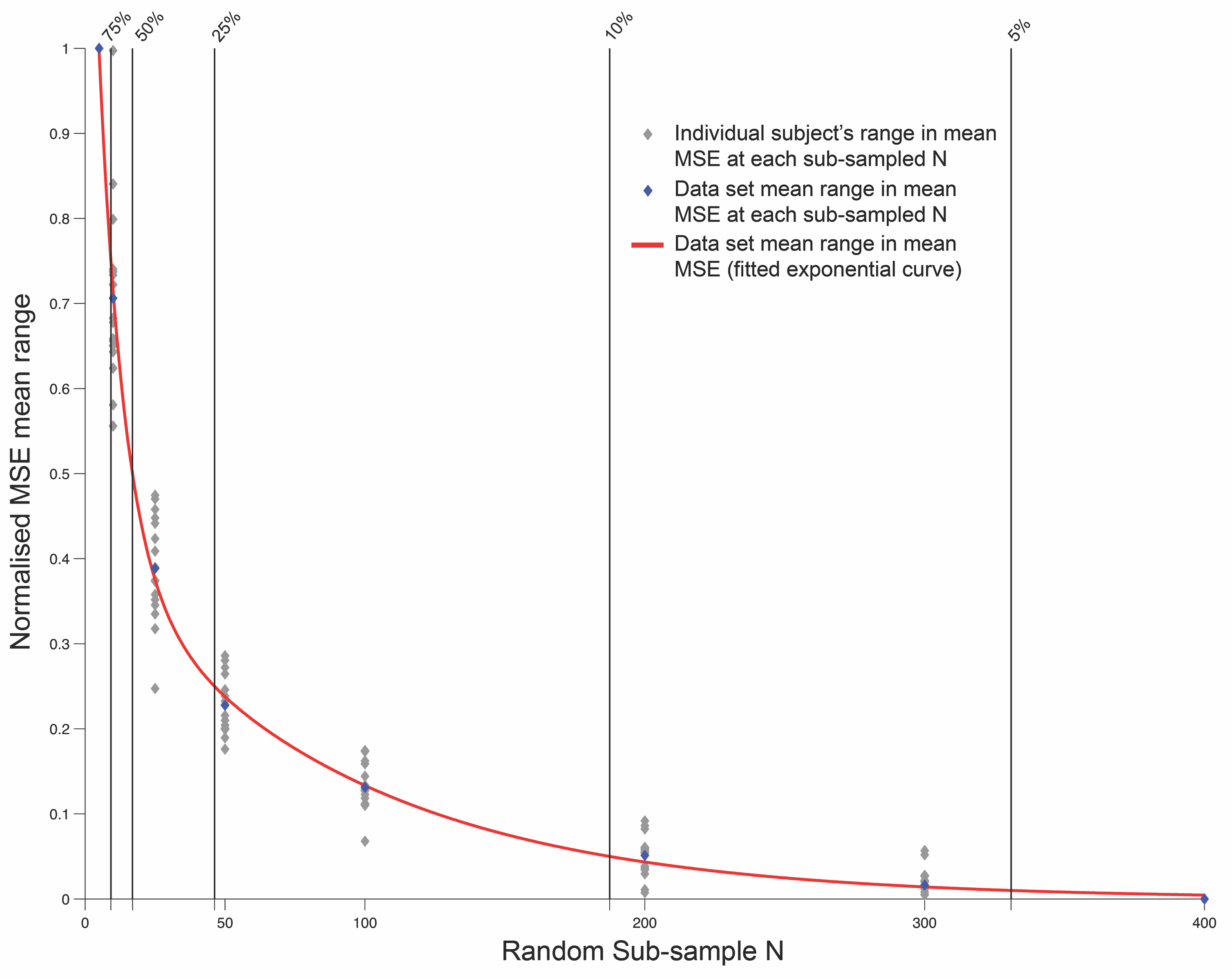


**Figure S5.** The relationship between subsample *N,* and the range in mean MSE at each subsample (given by 1000 randomly generated subsamples). The range in MSE is plotted as a percentage of mean MSE of the full dataset for each subject walking at 1.1ms-1. When sample size is *N* = >300 pressure records, then the range in mean MSE (given by the 1000 randomly generated subsamples) is less than 5% higher than the full dataset values for each subject. The observed range in mean MSE at smaller dataset sizes (*N* = 25 records) is more than 50% higher than the total dataset values observed for each subject.


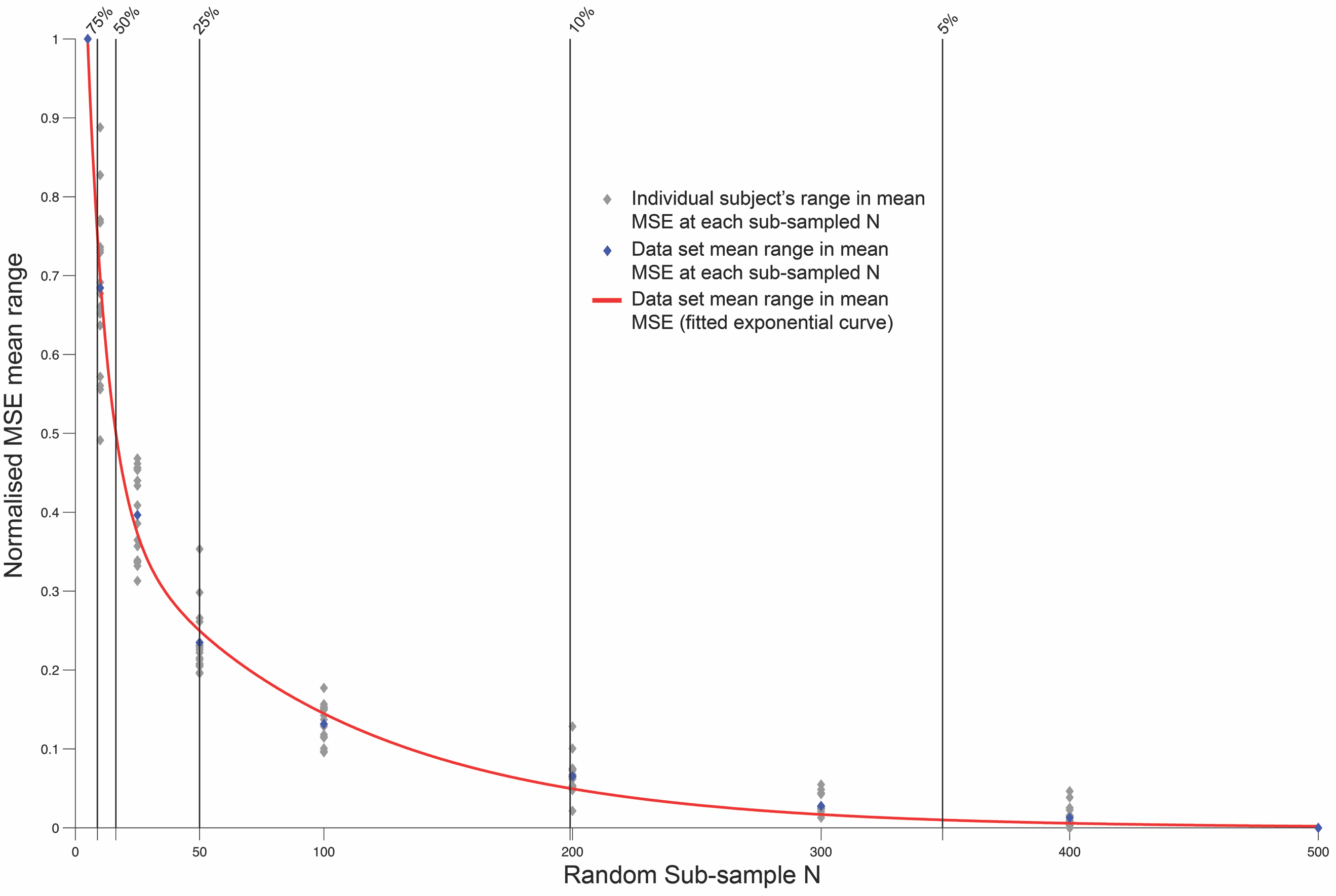


**Figure S6.** The relationship between subsample *N,* and the range in mean MSE at each subsample (given by 1000 randomly generated subsamples). The range in MSE is plotted as a percentage of mean MSE of the full dataset for each subject walking at 1.5ms-1. When sample size is *N* = >400 pressure records, then the range in mean MSE (given by the 1000 randomly generated subsamples) is less than 5% higher than the full dataset values for each subject. The observed range in mean MSE at smaller dataset sizes (*N* = 25 records) is more than 50% higher than the total dataset values observed for each subject.


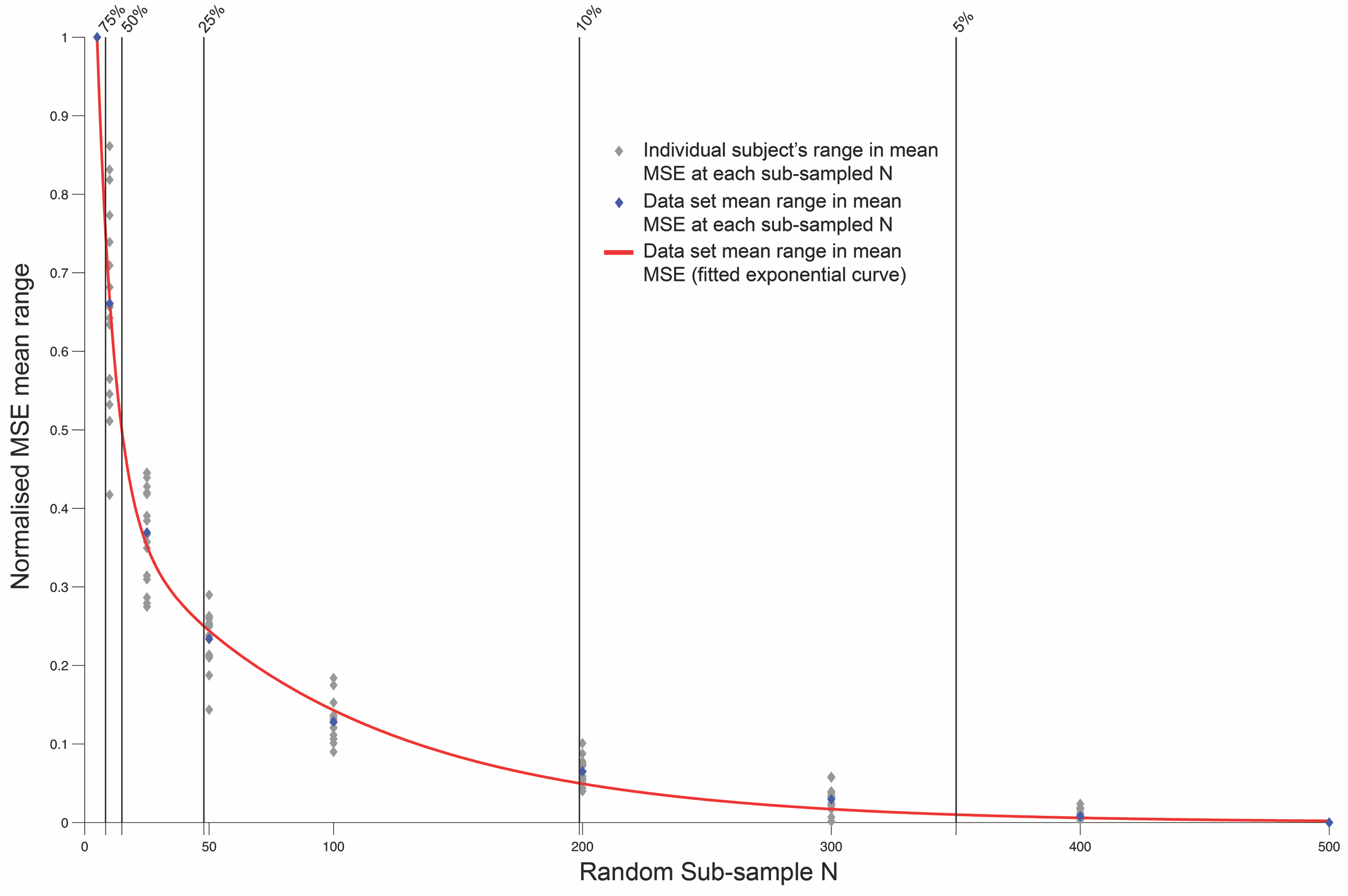


**Figure S7.** The relationship between subsample *N,* and the range in mean MSE at each subsample (given by 1000 randomly generated subsamples). The range in MSE is plotted as a percentage of mean MSE of the full dataset for each subject walking at 1.7ms-1. When sample size is *N* = >400 pressure records, then the range in mean MSE (given by the 1000 randomly generated subsamples) is less than 5% higher than the full dataset values for each subject. The observed range in mean MSE at smaller dataset sizes (*N* = 25 records) is more than 50% higher than the total dataset values observed for each subject.


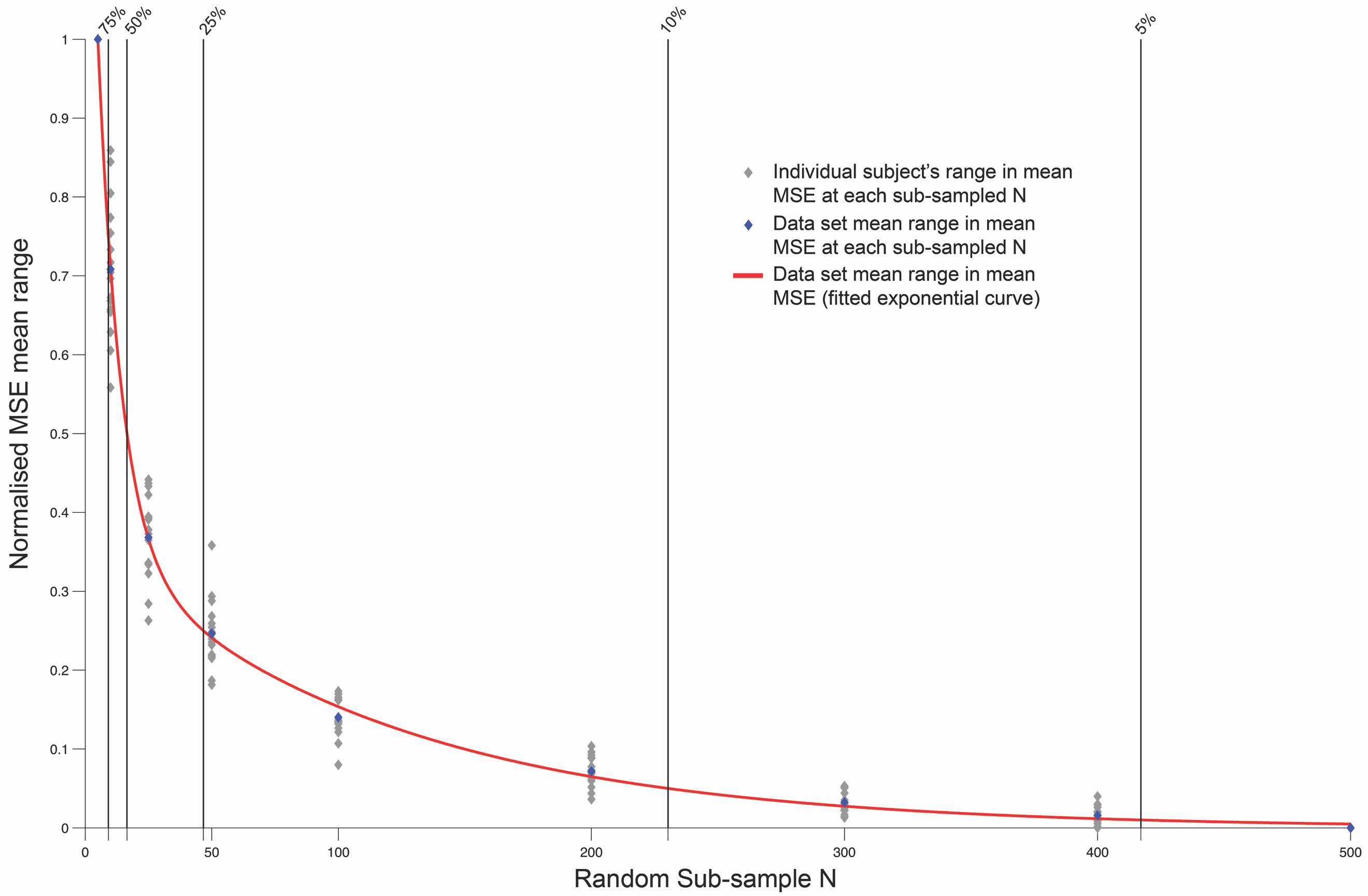


**Figure S8.** The relationship between subsample *N,* and the range in mean MSE at each subsample (given by 1000 randomly generated subsamples). The range in MSE is plotted as a percentage of mean MSE of the full dataset for each subject walking at 1.9ms-1. When sample size is *N* = >400 pressure records, then the range in mean MSE (given by the 1000 randomly generated subsamples) is less than 5% higher than the full dataset values for each subject. The observed range in mean MSE at smaller dataset sizes (*N* = 25 records) is more than 50% higher than the total dataset values observed for each subject.

**Table S1.** Number of records required to reach ‘reliability thresholds’ of 75%, 50%, 25%, 10% and 5% of the total sample MSE at each speed collected. Specifically, a reliability threshold of 5% indicates that the range in MSE values for 1000 subsampled populations at the *N* records is only 5% higher than the MSE of the full dataset *N*.

| Normalised MSE | *n* = 75% | *n* = 50% | *n* = 25% | *n* = 75% | *n* = 95% |
| --- | --- | --- | --- | --- | --- |
| Speed 1.1m/s | 9.21 (9) | 16.94 (17) | 46.26 (46) | 187.46 (187) | 330.9 (331) |
| Speed 1.3m/s | 8.82 (9) | 15.81 (16) | 46.55 (47) | 222.95 (223) | 401.35 (401) |
| Speed 1.5m/s | 8.88 (9) | 16.31 (16) | 49.95 (50) | 199.01 (199) | 348.88 (349) |
| Speed 1.7m/s | 8.42 (9) | 14.94 (15) | 47.92 (48) | 198.79 (199) | 350.1 (350) |
| Speed 1.9m/s | 9.13 (9) | 16.53 (17) | 46.69 (47) | 230.31 (230) | 417.16 (417) |

**Table 2.** Exponential equations describing the relationship between subsample *N* and the range of mean MSE from 1000 randomly generated subsamples.

| 1.1m/s | | Exponential equation | R^2^ |
| --- | --- | --- | --- |
| Least variable | f(x) = 2.284*exp(-0.05499*x) + 0.6421*exp(-0.001824*x) | 0.9706 |  |
| Mean | f(x) = 5.669*exp(-0.08487*x) + 1.884*exp(-0.003165*x) | 0.964 |  |
| Most variable | f(x) = 12.66 *exp(-0.08407 *x) + 2.547*exp(-0.002512*x) | 0.9349 |  |
| 1.3m/s |  |  |  |
| Least variable | f(x) = 3.704*exp(-0.05678*x) + 0.9245*exp(-0.002308*x) | 0.9784 |  |
| Mean | f(x) = 6.716*exp(-0.09162*x) + 2.065*exp(-0.002945*x) | 0.9528 |  |
| Most variable | f(x) = 10.47*exp(-0.05953*x) + 2.955*exp(-0.002236*x) | 0.9639 |  |
| 1. 5m/s |  |  |  |
| Least variable | f(x) = 3.465*exp(-0.1619*x) + 0.7651*exp(-0.003577*x) | 0.9307 |  |
| Mean | f(x) = 5.195*exp(-0.08164*x) + 1.642*exp(-0.002932*x) | 0.96 |  |
| Most variable | f(x) = 14.15*exp(-0.1665*x) + 3.389*exp(-0.003939*x) | 0.9589 |  |
| 1. 7m/s |  |  |  |
| Least variable | f(x) = 3.318*exp(-0.0551*x) + 1.085*exp(-0.0023*x) | 0.9769 |  |
| Mean | f(x) = 7.56*exp(-0.1257*x) + 2.104*exp(-0.003532*x) | 0.952 |  |
| Most variable | f(x) = 57.95 *exp(-0.2843*x) + 5.896*exp(-0.004846*x) | 0.9048 |  |
| 1.9m/s |  |  |  |
| Least variable | f(x) = 2.279*exp(-0.07063*x) + 0.7358*exp(-0.002994*x) | 0.9703 |  |
| Mean | f(x) = 5.566*exp(-0.08938*x) + 1.734*exp(-0.002981*x) | 0.955 |  |
| Most variable | f(x) = 8.125*exp(-0.1242*x) + 3.476*exp(-0.003983*x) | 0.9292 |  |
